# Enhancing Icephobic Coatings: Exploring the Potential of Dopamine-Modified Epoxy Resin Inspired by Mussel Catechol Groups

**DOI:** 10.3390/biomimetics9060349

**Published:** 2024-06-08

**Authors:** Mohammad Sadegh Koochaki, Gelareh Momen, Serge Lavoie, Reza Jafari

**Affiliations:** 1 Département des Sciences Appliquées, Université du Québec à Chicoutimi, Chicoutimi, QC G7H 2B1, Canada; mohammadsadegh.koochaki1@uqac.ca (M.S.K.); reza_jafari@uqac.ca (R.J.); 2 Département des Sciences Fondamentales, Université du Québec à Chicoutimi, Chicoutimi, QC G7H 2B1, Canada; serge_lavoie@uqac.ca

**Keywords:** icephobic coating, epoxy, dopamine, ice adhesion, quasi-liquid layer, low-temperature ATR-FTIR

## Abstract

A nature-inspired approach was employed through the development of dopamine-modified epoxy coating for anti-icing applications. The strong affinity of dopamine’s catechol groups for hydrogen bonding with water molecules at the ice/coating interface was utilized to induce an aqueous quasi-liquid layer (QLL) on the surface of the icephobic coatings, thereby reducing their ice adhesion strength. Epoxy resin modification was studied by attenuated total reflectance infrared spectroscopy (ATR-FTIR) and nuclear magnetic resonance spectroscopy (NMR). The surface and mechanical properties of the prepared coatings were studied by different characterization techniques. Low-temperature ATR-FTIR was employed to study the presence of QLL on the coating’s surface. Moreover, the freezing delay time and temperature of water droplets on the coatings were evaluated along with push-off and centrifuge ice adhesion strength to evaluate their icephobic properties. The surface of dopamine-modified epoxy coating presented enhanced hydrophilicity and QLL formation, addressed as the main reason for its remarkable icephobicity. The results demonstrated the potential of dopamine-modified epoxy resin as an effective binder for icephobic coatings, offering notable ice nucleation delay time (1316 s) and temperature (−19.7 °C), reduced ice adhesion strength (less than 40 kPa), and an ice adhesion reduction factor of 7.2 compared to the unmodified coating.

## 1. Introduction

Ice adhesion and accretion on exposed surfaces are widespread and inevitable phenomena that occur naturally during different types of precipitation, such as frosting, condensation freezing, and frozen rain. These issues lead to irreparable equipment damage, economic losses, and safety concerns across various domains, including aviation, wind energy, solar power, power transmission lines, vehicles, marine vessels, and offshore oil platforms [1]. Over the years, numerous active and passive techniques and materials have been developed to prevent, mitigate, or endure icing and its associated consequences [2]. However, traditional active de-icing methods have drawbacks, including high energy consumption, low efficacy, and negative environmental impact [3]. On the other hand, the term “anti-icing” commonly refers to passive systems, predominantly in the form of icephobic coatings that operate without the need for external energy input [4,5]. In this regard, various passive icephobic coatings have been developed, including superhydrophobic surfaces (SHS) [6], self-lubricating layers and slippery liquid-infused surfaces [7,8], low elastic modulus gels and elastomers [9,10,11,12], and multiscale crack initiator-promoted surfaces [5].

Passive anti-icing methods have made significant advancements in reducing ice accretion and adhesion strength. However, they still encounter challenges regarding mechanical durability and maintaining low ice adhesion strength during icing and de-icing cycles, which limits their widespread industrial applications [13,14]. Silicon elastomers have emerged as promising base materials among the numerous icephobic surfaces and coatings in published literature, owing to their chemical inertness, tunable mechanical properties, hydrophobicity, and moldability [15]. High deformation incompatibility (DI) of silicone elastomers with ice is the key parameter leading to their low ice adhesion strength. However, this characteristic highly relies on the softness of the silicone elastomer, which usually leads to weaker mechanical properties and durability [16,17]. In contrast, epoxy resins have found extensive use in numerous industrial applications, such as anti-corrosion coatings, adhesives, and composites, owing to their favorable physico-mechanical properties, exceptional chemical and thermal resistance, durability, and processability [18]. Therefore, epoxy-based polymers have also gained attention to be utilized for developing robust and durable icephobic coatings [19].

Fluoropolymers [20], perfluorosilanes, aminosilanes [21,22], polytetrafluoroethylene (PTFE) particles [23], and also polydimethylsiloxane (PDMS) [24] have been utilized to enhance the icephobic properties of epoxy-based coatings. Decreasing the surface energy by introducing silane/fluorosilane-containing additives has been reported for the development of icephobic epoxy coatings. However, these studies either did not report the ice adhesion strength or did not adequately assess the durability of the system [21,22]. Similarly, in another approach, PTFE particles were sprayed onto the surface of partially-cured epoxy resin to create an icephobic coating. Although the evolution of ice adhesion strength over 20 icing/de-icing cycles was examined, the weak interfacial interactions between PTFE and epoxy resin, as well as their impact on particle stability and the durability of icephobicity were not investigated [23]. Another method involved grafting fluorine-containing chains onto the epoxy resin to produce low surface energy icephobic coatings. The ice adhesion strength for the most stable modified sample was found to be around 100 kPa before and after abrasion with sandpaper [20].

In contrast to the previously mentioned approaches that primarily focus on reducing surface energy and enhancing hydrophobicity, a different approach based on the hydrophilicity of surfaces has gained significant attention in recent years for the development of icephobic surfaces. Inspired by ice skating, these surfaces, known as self-lubricating surfaces or aqueous lubricating layers, rely on the presence of a non-frozen liquid-like water layer that adheres to hygroscopic molecules or polymers, acting as a lubricating layer that facilitates the detachment of ice from the surface [25,26]. In this context, various hydrophilic polymers have been developed to create icephobic coatings with aqueous lubricating layers, including hyaluronic acid-dopamine [25], poly(acrylic acid) [27], poly(acrylic acid)-dopamine [28], and polydimethylsiloxane-polyethylene glycol (PEG-PDMS) copolymers [7].

The use of dopamine is a well-established strategy for modifying materials with mussel-inspired catechol groups in different applications [29]. The two bidentate hydroxyl groups on the aromatic ring of catechol induce remarkably strong hydrogen bonding with a lifetime approximately 10^8^ times longer than that of hydrogen bonding between water molecules [30]. This characteristic imparts unique properties to catechol-containing materials, such as high adhesion strength, wet surface bonding, and self-healing. In the present research, this unique property of catechol groups was utilized for the first time in the development of a robust epoxy-based resin tailored for anti-icing applications. The structure of the epoxy-based resin was modified using dopamine molecules to enhance its hydrophilic properties. The strong affinity of grafted catechol groups for hydrogen bonding with water molecules at the ice/coating interface was employed to induce an aqueous lubricating layer on the surface of the icephobic coatings, thereby reducing their ice adhesion strength.

## 2. Materials and Methods

### 2.1. Materials

Dopamine hydrochloride (dopamine precursor) and sodium hydroxide were purchased from Sigma-Aldrich (St. Louis, MO, USA) and Laboratoire MAT (Québec, QC, Canada), respectively. Solvents including dichloromethane and methanol were purchased from Fisher Scientific (Ottawa, ON, Canada). These materials were used as received to modify a hydrogenated diphenylpropane epoxy resin (Eponex^®^ 1510, Epoxy equivalent weight = 210 g/eq) provided by Hexion (Columbus, OH, USA). Both modified and pristine epoxy resins were cured by 1,8-Diamino-3,6-dioxaoctane (JEFFAMINE^®^ EDR-148, Amine hydrogen equivalent weight = 37 g/eq) provided by Huntsman Corporation (The Woodlands, TX, USA). Methyl Ethyl Ketone (MEK) was also purchased from Fisher Scientific and used as the solvent for applying the coatings to the substrate.

### 2.2. Synthesis of Dopamine-Modified Epoxy Resin

**Figure 1:**
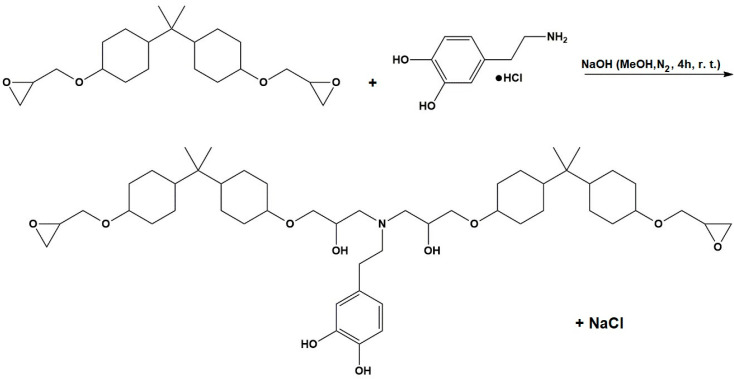
Synthesis of the dopamine-modified epoxy resin.

The dopamine-modified epoxy resin for icephobic coating applications was synthesized using the epoxide ring-opening reaction illustrated in Figure 1. Initially, an excess of epoxy resin (16.35 g, 39 mmol) and dopamine hydrochloride (2.5 g, 13 mmol) were dissolved in 250 mL of methanol in a 500 mL two-neck round bottom flask. The mixture was bubbled with nitrogen during the mixing for 60 min to maintain an inert atmosphere. To control the degree of polymerization and promote the reaction of dopamine’s amine groups, the molar ratio between the epoxy resin and dopamine hydrochloride was set at 3:1. Similarly, NaOH (0.5 g, 13 mmol) was dissolved separately in 100 mL of methanol under a nitrogen atmosphere. Afterward, the NaOH solution was added dropwise to the reaction mixture, enabling the progressive activation of the dopamine-NH_2_ groups by neutralizing the hydrochloric acid molecules. The resulting mixture was stirred for 4 h at room temperature under a nitrogen atmosphere to allow a complete reaction between the reagents. Subsequently, methanol was removed from the mixture by a vacuum evaporator, and the crude product was dissolved in the minimum amount of dichloromethane. This step facilitated the precipitation and filtration of NaCl, which formed as a result of the neutralization reaction between HCl and NaOH. Finally, dichloromethane was removed under reduced pressure to obtain the catechol-modified epoxy resin.

### 2.3. Coatings Preparation and Application

**Figure 2:**
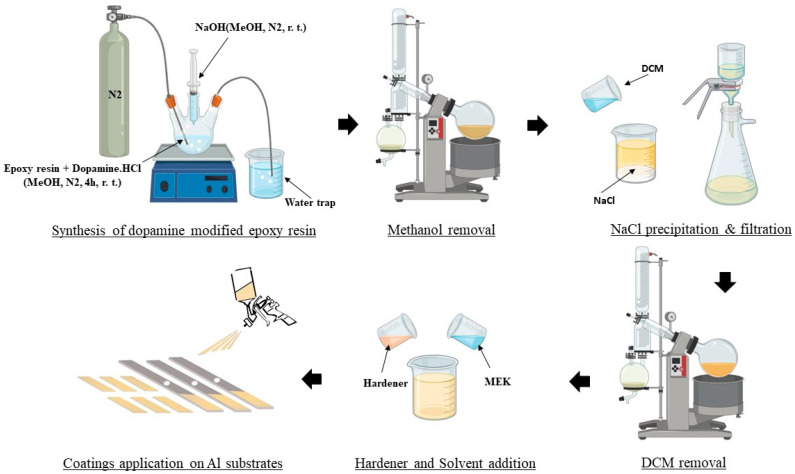
Schematic of the epoxy coating modification and application process.

The pristine epoxy resin (Eponex^®^ 1510) was mixed with the hardener (JEFFAMINE^®^ EDR-148) at a stoichiometric (100 to 17.6) weight ratio to be assessed as the control coating. On the other hand, the modified epoxy resin was mixed with the same hardener according to its nominal measured epoxy equivalent weight (496 g/eq) at a 100 to 7.4 weight ratio. Both mixtures were then diluted with 25% *w*/*w* of MEK to achieve adequate viscosity for spray coating. The mixtures were then immediately applied on previously sanded (800 SiC paper) and degreased (acetone) substrates including 6061-T6 alloy aluminum panels and the Centrifugal Adhesion Test (CAT) beams [31] using an air spray gun. The wet film thickness was adjusted to achieve a 100 ± 10 µm dry film thickness. The applied coatings were left to cure at room temperature for 7 days before being tested. A brief schematic of the epoxy coating modification and application process is presented in Figure 2.

### 2.4. Characterizations

#### 2.4.1. Characterization of Dopamine-Modified Epoxy Resin

The monitoring of the epoxide ring opening reaction with amine groups of dopamine was carried out using Fourier transform infrared spectroscopy (FTIR) in attenuated total reflection mode, utilizing a Spectrum II spectrometer (Perkin Elmer, Waltham, MA, USA) within the infrared range of 4000–650 cm^−1^. The reaction mixture solution was sampled before and after neutralization with NaOH for testing purposes. A droplet of each sample was placed on the crystal of the device and left to sit for 15 min before testing to allow for methanol evaporation.

Additionally, the structure of the dopamine-modified epoxy resin was investigated using nuclear magnetic resonance (NMR) spectroscopy. Proton NMR spectra were recorded using a Bruker Ascend 500 MHz NMR spectrometer (Bruker, Milton, ON, Canada) in DMSO-*d*_6_ as the solvent to confirm the success of the reaction. Chemical shifts were expressed in parts per million (ppm) relative to the residual solvent peak (2.50 ppm).

#### 2.4.2. Surface Characterization of the Coatings

X-ray photoelectron spectroscopy (XPS) analysis (PHI 5600-ci spectrometer, Physical Electronics, Chanhassen, MN, USA) was conducted on the prepared coatings to assess their surface chemical composition. Analysis was performed on a 0.5 mm^2^ area using a standard achromatic Al X-ray source with a maximum energy of 1486.6 eV. Spectra were acquired at an incidence angle of 45° with respect to the normal surface. High-resolution spectra were obtained using a standard magnesium X-ray source without neutralization.

The water contact angle (WCA) and contact angle hysteresis (CAH) on the surface of the prepared coatings were measured at 25 °C using a Kruss™ DSA100E goniometer (KRÜSS Scientific, Hamburg, Germany). For WCA measurements, a 4 µL water droplet was placed on the surface, and the contact angle was recorded over time. CAH was measured as the difference between the advancing and receding contact angles of a 35 µL droplet of water sliding on the coatings’ surface while tilting. To determine the surface roughness of the samples, a 3D optical profilometer (Profil3D Filmetrics, San Diego, CA, USA) was utilized. The measurements were conducted using white light interferometry (WLI) to capture surface profiles and assess roughness. The phase shifting interferometry (PSI) mode was employed, allowing for roughness measurements with a precision of 0.001 µm for the samples.

#### 2.4.3. Mechanical Properties of the Coatings

Different evaluations were used to study the mechanical properties of the prepared coatings. A pendulum hardness tester (byko-swing Persoz, Byk-Gardner, Columbia, MD, USA) was used to study the hardness of the applied coatings according to ASTM D4366 [32]. The adhesion strength of the coatings to aluminum substrates was tested by the pull-off adhesion test method according to ASTM D4541 (PosiTest AT-T, DeFelsko, Ogdensburg, NY, USA) [33]. The surface of aluminum substrates utilized for the pull-off adhesion test was prepared before the coatings’ application using the atmospheric-pressure plasma treatment method (Plasma Jet AS400, Plasmatreat GmbH, Steinhagen, Germany) [34]. To study the abrasion resistance of the samples, a Taber abrasion test (TABER^®^ Rotary Platform Abrasion Tester, Model 1700, TABER Industries, North Tonawanda, NY, USA) was also performed according to ASTM D4060 with CS17 wheels for 1000 cycles and 1000 g load weight [35]. The tensile strength and elongation at break of the prepared coatings were also studied according to ASTM D2370 [36]. In this regard, both modified and pristine epoxy resins were mixed with the hardener in stoichiometric ratios and cast in polypropylene molds with rectangular cavities with dimensions of 100 mm × 13 mm × 0.5 mm. After 7 days of curing at room temperature, the prepared free films were removed from the molds and tested at a 5 mm/min elongation rate by a tensile apparatus (TA.TX Plus100C, Stable Micro System, Godalming, UK). All experiments were replicated 5 times under standard laboratory conditions to ensure result accuracy.

#### 2.4.4. Icephobic Properties of the Coatings

Differential scanning calorimetry (DSC Q250, TA Instruments, New Castle, DE, USA) was employed to investigate the evolution of ice nucleation temperature on the surface of prepared coatings. Thin layers (approximately 100 µm) of the coatings were applied into Tzero pans and allowed to cure at room temperature for 7 days before testing. Subsequently, a 5 mg water droplet was placed in each pan, followed by sealing with a lid. DSC measurements were then conducted on the samples, ranging from +30 °C to −40 °C at a rate of 5 °C/min. Moreover, the freezing delay time of the samples was assessed using a Kruss™ DSA100E goniometer equipped with a cold chamber featuring a Peltier base plate with a controllable temperature unit. Experiments were conducted at −20 °C, with the coatings left in the chamber for 30 min before testing to ensure they sufficiently cooled down. The freezing delay time was determined by measuring the time it took for a 4 µL water droplet to freeze on the surface of the coatings at −20 °C. To minimize the influence of atmospheric moisture on the testing conditions, the cold chamber was purged with N_2_ during the evaluations.

**Figure 3:**
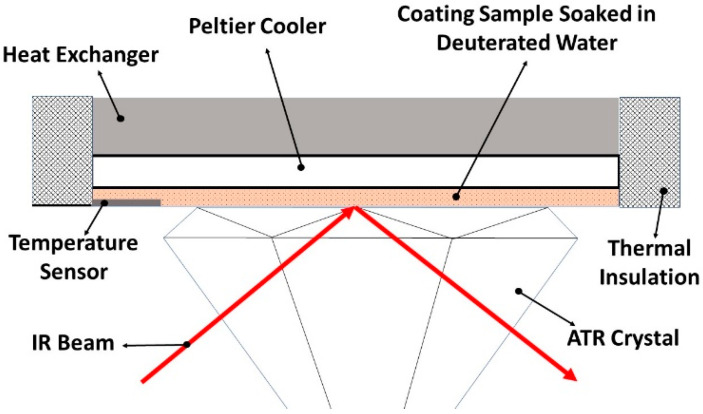
Schematic illustration of the cold-based apparatus utilized to conduct low-temperature ATR-FTIR spectroscopy.

ATR-FTIR spectroscopy (Spectrum II, Perkin Elmer, Waltham, MA, USA) was employed to investigate the coating–water interface at low temperatures and to examine the presence of QLL on the coatings’ surface. A cold-base apparatus (Figure 3) was utilized to regulate the temperature of the coatings on the ATR prism during spectroscopy. The temperature controller sensor (accuracy = 0.1 °C) was positioned at the interface of the coating and prism to ensure precise measurement and control of the interface temperature. According to the interference expected by overlapping the absorption bands of the amine groups of the coatings with the hydroxyl groups of water, deuterium oxide (D_2_O, 99.9%, CDN ISOTOPES INC., Pointe-Claire, QC, Canada) was substituted for water during spectroscopic analysis. The O-D stretching band of deuterium oxide at 2467 cm^−1^ does not interfere with any of the coatings and notably shifts to 2355 cm^−1^ upon freezing. The substitution of water with D_2_O has minimal impact on the experimental results, as the freezing behavior of D_2_O closely resembles that of H_2_O due to their similar physical and chemical properties. Free films of the coating samples (100 ± 10 µm thickness) were immersed in D_2_O for 6 h before FTIR analysis. The FTIR data were recorded continuously (every 6 s) from +25 °C to −30 °C at a rate of 1 °C/min using PerkinElmer’s Spectrum^TM^ TimeBase software (Application Version: 10.7.2.1630) within the infrared range of 4000–400 cm^−1^.

The ice adhesion strength of the prepared coatings was assessed using both the push-off and centrifuge adhesion test (CAT) methods. In the push-off test, cylindrical polyethylene molds with an inner diameter of 1.3 cm were placed onto the surface of the coatings and allowed to cool down at −10 °C in a cold chamber for 2 h. Five samples were prepared for each coating. Subsequently, the molds were filled with deionized water and left to freeze, forming ice cylinders on the coating surface. The samples were then kept in the cold chamber at −10 °C for 24 h before testing. To measure the force required to detach the ice cylinders from the coating surface, a digital force gauge (FG-3005, Shimpo Instruments, Lynbrook, NY, USA) was utilized. The ice adhesion strength was calculated by dividing the maximum force required for ice detachment by the coated area exposed to ice. Additionally, the push-off test was repeated on the samples for ten consecutive icing/de-icing cycles to assess the durability of their icephobic properties.

For conducting the CAT test, six aluminum beams (6061-T6 alloy, 340 mm × 31.8 mm × 6.4 mm) were prepared for each coating. The coatings were sprayed overtop of one end of the beams, while the other end was kept blank to maintain balance after icing. Coated beams were left in the lab for 7 days to allow the coatings to become fully cured. The beams were then placed in a cold room to reach a temperature of −8.0 °C ± 0.1 and simultaneously iced for about 35 min by spraying supercooled water droplets (mean volumetric diameter = 324 µm). In this regard, an area of 1100 ± 70 mm^2^ from each beam was subjected to precipitation resulting in 5.5 ± 0.5 g of ice (7 ± 1 mm thickness). Each beam was tested in the centrifuge VAT (Vortex Airflow Tunnel) individually at −10.0 °C ± 0.1, rotating from zero to ice detachment speed with constant acceleration of 300 rpm/s. The centrifugal force applied to the ice was calculated using Equation (1), which considers the mass of the ice (*m*), the beam radius (*r*), and the rotation speed at which ice shedding occurs (*w*):

(1) $$F=mr\omega^{2},$$

The centrifugal ice adhesion strength was then calculated by dividing the ice centrifugal force by the surface area covered by the ice.

## 3. Results and Discussion

### 3.1. Chemical Characterization of the Modified Epoxy Resin

#### 3.1.1. FTIR

**Figure 4:**
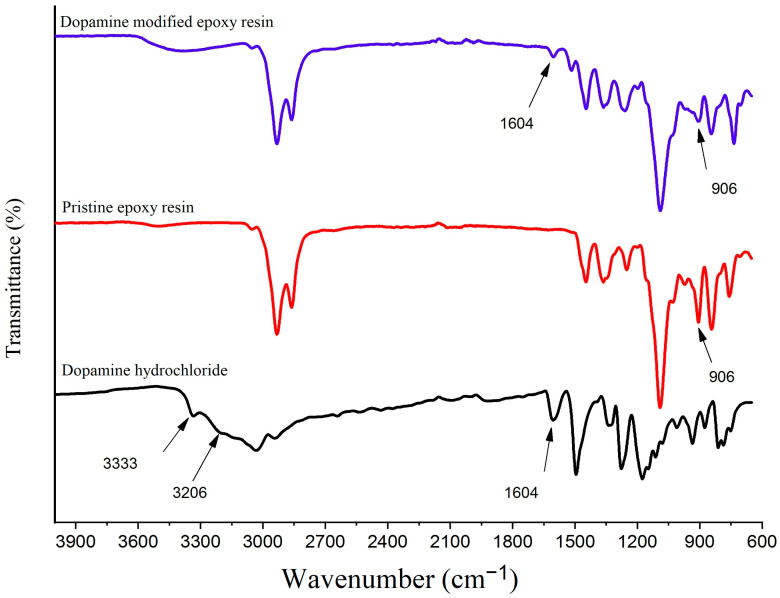
The FTIR spectrum of dopamine hydrochloride, pristine epoxy resin, and dopamine-modified epoxy resin.

The FTIR spectra analysis of dopamine hydrochloride, pristine epoxy resin, and dopamine-modified epoxy resin is depicted in Figure 4.

The spectra reveal distinct features for each compound. In the case of dopamine hydrochloride, the bands at 3333 cm^−1^ and 3206 cm^−1^ correspond to the stretching vibrations of the primary amine group, while the band at 1604 cm^−1^ can be attributed to its bending vibrations [37,38]. For the other compound, pristine epoxy resin, the stretching vibration band of the oxirane group can be seen at 906 cm^−1^ [39]. Upon the introduction of dopamine’s amine groups, which induces the opening of the epoxide ring, the modified epoxy resin displays characteristic peaks of both dopamine and the epoxy resin, along with their evolutions throughout the favored reaction. Notably, in the case of the dopamine-modified epoxy resin (DEP), the bands at 3333 cm^−1^ and 3206 cm^−1^ associated with the N-H stretching vibrations cannot be identified, indicating a change in the chemical environment. Additionally, a significant decrease in the intensity of the bands at 1604 cm^−1^ (representing the primary amine bending vibrations) and 906 cm^−1^ (corresponding to the epoxide ring stretching vibrations) is observed. The disappearance of the N-H stretching bands and the decrease in intensity of both the primary amine bending vibrations and the epoxide ring stretching vibrations provide evidence of a successful modification reaction [40].

#### 3.1.2. NMR

**Figure 5:**
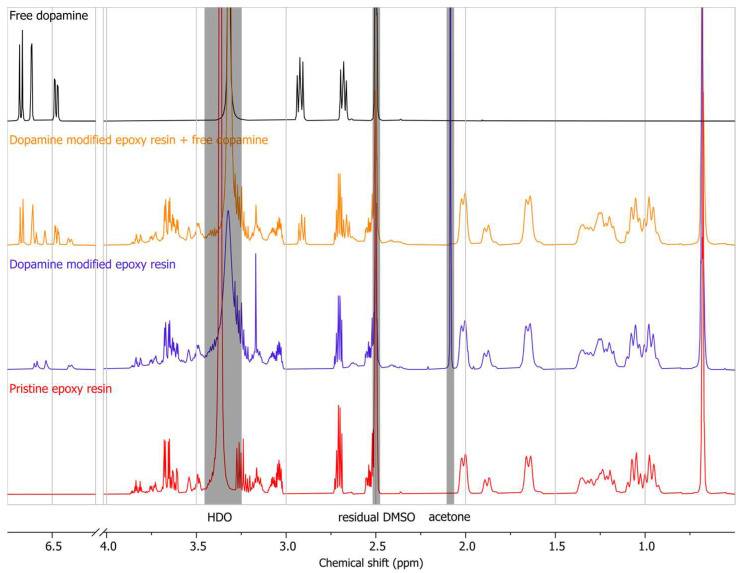
^1^H NMR spectrum (DMSO-*d*_6_, 500 MHz) of free dopamine, the mixture of dopamine-modified epoxy resin and free dopamine, dopamine-modified epoxy resin, and pristine epoxy resin.

The ^1^H NMR spectrum of dopamine hydrochloride, the modified epoxy resin, an equimolar mixture of them (theoretically calculated), and the pristine epoxy resin are depicted in Figure 5. The chemical shifts attributed to the aromatic protons of the dopamine molecule can be identified ranging from 6.69 to 6.46 ppm in its ^1^H NMR spectrum [40], while no signal within this region was recorded for the pristine epoxy resin. The hydrogenated epoxy resin selected for the present research facilitated the detection of aromatic protons introduced by dopamine molecules into the structure of the modified polymer. Signals associated with the protons of the epoxy resin’s cyclohexane rings are also observed in the 2.10–0.88 ppm region, while the methyl groups are at 0.68 ppm. Other signals in the mid-field region (3.90–2.30 ppm) mainly correspond to aliphatic protons associated with heteroatoms (–CH_2_N, –CH_2_O, and –CHOH) of the epoxy resin, as well as methylene groups of dopamine. The modified epoxy resin, similar to pure dopamine, exhibits aromatic protons belonging to the catechol group. However, a slight shift is observed for the aromatic protons of the modified epoxy resin when compared to pure dopamine. This shift can be attributed to the formation of a covalent bond between dopamine and the hydrogenated epoxy resin, resulting in the modified structure. To ensure that this shift was not due to the concentration or non-covalent interaction of the dopamine and epoxy resin, an equimolar mixture of dopamine hydrochloride and the modified epoxy resin was analyzed. The resulting spectrum demonstrated the presence of two sets of aromatic protons corresponding to both free dopamine hydrochloride and the modified epoxy resin. This coexistence confirms that the observed aromatic chemical shifts in the modified epoxy resin are indeed attributed to the grafted dopamine molecules, providing further evidence of the successful biomimetic modification reaction.

### 3.2. Surface Analysis of the Prepared Coatings

#### 3.2.1. XPS

**Figure 6:**
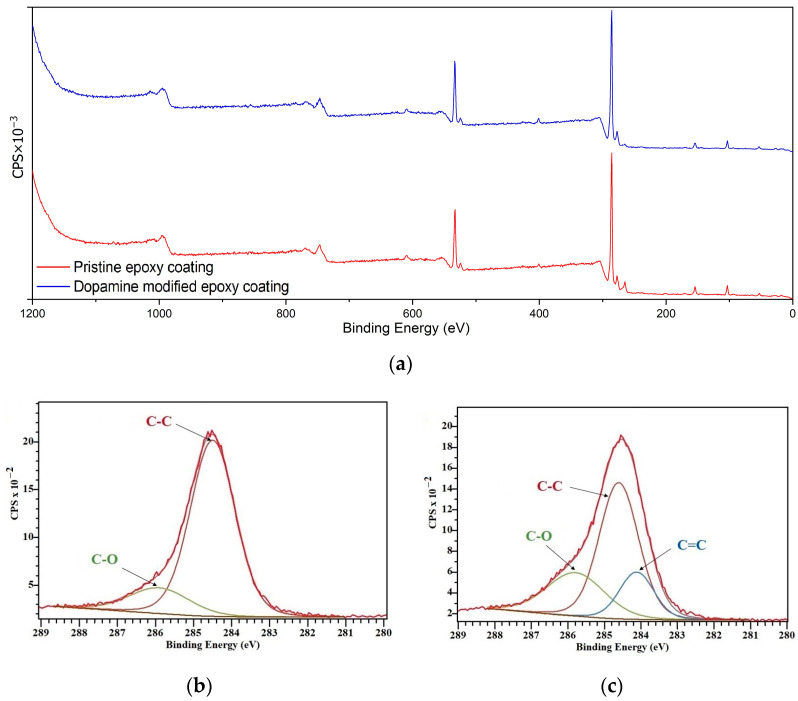
XPS results: (**a**) XPS survey spectra, (**b**) high-resolution C 1s spectra of the pristine epoxy coating, and (**c**) high-resolution C 1s spectra of the dopamine-modified epoxy coating.

XPS analysis was conducted on the prepared coatings to assess the chemical composition of their surface, providing insights into the role of surface chemistry in their icephobic properties [41]. Figure 6 illustrates the XPS spectra of the pristine and dopamine-modified coatings. Peaks corresponding to carbon (C 1s), nitrogen (N 1s), and oxygen (O 1s) were detected at binding energies around 284, 401, and 533 eV, respectively. All binding energy values were calibrated using the reference peak of C 1s at 284.8 eV. Although the XPS survey spectra of the coatings (Figure 6a) exhibited minimal differences due to similar elemental compositions and closely related chemical structures, high-resolution spectra of C 1s were analyzed to investigate the surface chemical composition.

According to the curve fittings of C 1s for the pristine epoxy coating (Figure 6b), the peaks at 284.5 eV and 285.9 eV correspond to the C–C and C–O moieties, respectively [42]. For the dopamine-modified coating (Figure 6c), an additional peak at 284.1 eV corresponding to C=C is observed, in addition to peaks corresponding to C–C (284.6 eV) and C–O (285.8 eV) moieties, which can be attributed to the aromatic C=C bonds of the catechol units [43]. Moreover, according to the relative area of the peaks measured by Casa XPS software (version: 2.3.26PR1.0) which are presented in Table 1, the concentration of C–O bands on the surface increased from 16.35% for the pristine epoxy coating to 26.34% for the dopamine-modified one, which is a plausible result of introducing bidentate hydroxyl groups of dopamine via the acquired modification reaction.

#### 3.2.2. Wettability of the Prepared Coatings

**Figure 7:**
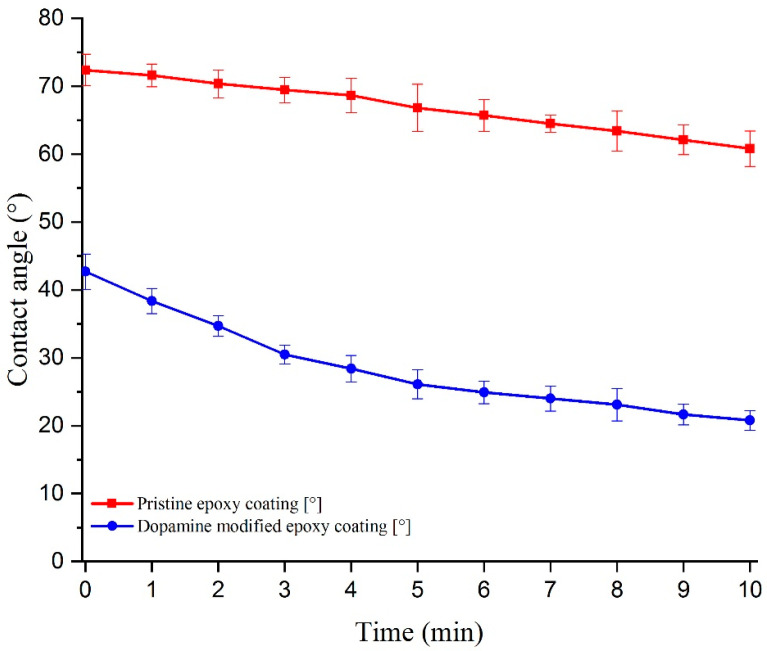
WCA evolution over time for the pristine and modified epoxy coatings.

**Figure 8:**
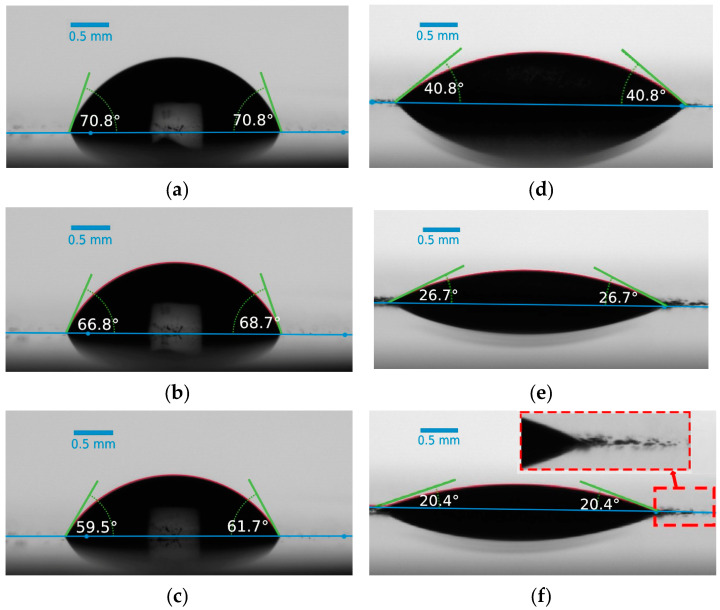
Water droplet evolution at the initial dripping moment (t = 0), as well as after 5 and 10 min for the pristine ((**a**), (**b**), and (**c**) respectively) and modified ((**d**), (**e**), and (**f**) respectively) epoxy coatings.

The investigation of WCA and CAH on the prepared coatings was conducted to assess the surface’s wettability. WCA and CAH measurements provide insights into how water droplets interact with the surface and can indicate the hydrophilic or hydrophobic nature of the coatings. The ability of a surface to induce a liquid-like interfacial water layer is in direct relation with its affinity to water droplets and hydrophilicity [7]. Figure 7 illustrates the evolution of the water contact angle over time for both the pristine epoxy and dopamine-modified epoxy coatings. The initial WCA for the pristine epoxy coating was approximately 72.4°, while for the modified coating, it reduced significantly to 42.7°. This notable decrease in WCA can be directly attributed to the presence of catechol units on the surface and their strong hydrogen bonding interactions with water molecules [44,45,46]. Furthermore, the WCA/time measurement curves indicate that the reduction in WCA, especially within the initial 5 min, was more rapid for the dopamine-modified coating compared to the unmodified one, resulting in a higher overall decrease within the 10 min evaluation period (reduction of 11.6° ± 3.5° and 21.9° ± 3.0° for the pristine and modified epoxy coatings, respectively). The gradual decrease in WCA for the pristine sample can be attributed to water evaporation and the consequent decrease in droplet volume [47]. To better illustrate the effects of dopamine grafting on the wettability of the prepared coatings, images of water droplets on the coatings were captured at the initial dripping moment (t = 0), as well as after 5 and 10 min (Figure 8). In the case of the modified coatings, the affinity of water to spread over the surface is evident, as small droplets can be observed detaching from the edges of the main droplet. This phenomenon, resulting from the strong hydrophilic nature of the catechol units present in the modified epoxy resin, is magnified in Figure 8f for better visualization. Moreover, water contact angle hysteresis measurements demonstrated a CAH of approximately 12° for the pristine epoxy coating and 20° for the dopamine-modified epoxy coating, at tilting angles of 13.3° and 20.2°, respectively. The elevated CAH in the dopamine-modified epoxy coating provides additional evidence of its heightened affinity for water, demanding greater force to displace the contact line of the water droplet on the surface [48].

### 3.3. Mechanical Properties of the Prepared Coatings

The effect of dopamine grafting on the mechanical properties of the epoxy coating was studied by different methods. In the results presented in Table 2, it was observed that the tensile strength of the modified epoxy coating decreased from 20.8 ± 1.6 MPa to 9.6 ± 0.9 MPa. In contrast, the elongation at break of the coating increased from 9 ± 3% to 45 ± 8%. The elastic modulus, determined from the stress–strain curves, exhibited a similar trend to the tensile strength, indicating the evolution of the coatings’ stiffness as a result of the dopamine modification reaction. This phenomenon can be attributed to the introduction of dopamine molecules into the structure of the epoxy resin leading to a reduction in the number of epoxide groups available for crosslinking reactions with the amine hardener. As a result, the crosslinking density of the modified epoxy coating decreases. This decrease in crosslinking density contributes to lower tensile strength, as there are fewer intermolecular connections to withstand applied forces. Additionally, the reduced crosslinking density allows for easier movement of the molecular chains within the coating. This increased mobility enhances the coating’s flexibility, resulting in a higher elongation at break [49,50]. The pendulum hardness results exhibited a similar trend to the tensile strength results, showing a decrease of approximately 50% compared to the original amount. This correlation can be attributed to the fact that both parameters, pendulum hardness and tensile strength, are directly influenced by the crosslink density of the material. In addition to the changes observed in other mechanical properties, the abrasion resistance of the dopamine-modified epoxy coating showed a slight decrease, consistent with the overall trend. It is important to note that despite the reduction in mechanical properties, the coating still maintains an acceptable condition compared to other icephobic coatings [51,52,53]. The pull-off adhesion strength results of the dopamine-modified coatings on aluminum substrates showed a notable increase in comparison with the pristine epoxy coatings. As can be seen in Table 2, introducing dopamine in the structure of epoxy resin led to a 71.8% increase in its adhesion to the substrate. This improvement can be attributed to the strong hydrogen bonding of catechol groups and their co-ordination bonds with metal oxide surfaces [54].

### 3.4. Icephobic Properties of the Prepared Coatings

#### 3.4.1. Freezing Delay Temperature and Time

**Figure 9:**
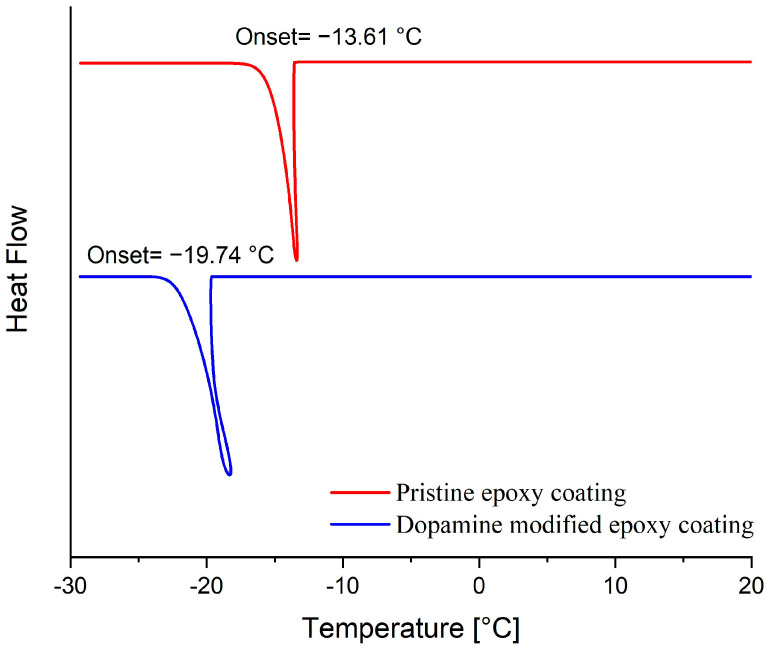
DSC thermograms presenting the ice nucleation temperature on the surface of pristine and dopamine-modified epoxy coatings.

**Figure 10:**
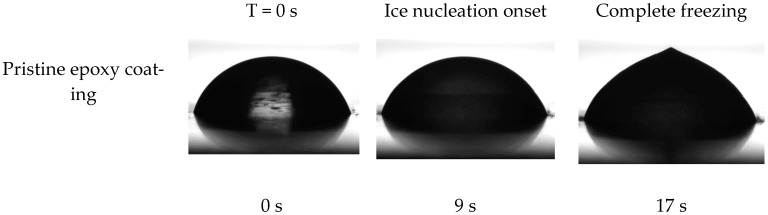

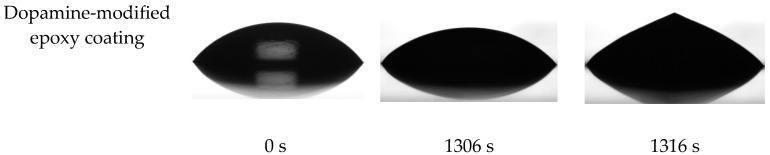
Droplet shape and freezing delay time of water droplet on the surface of prepared coatings at −20 °C.

The ice nucleation temperature and freezing delay time of water droplets on the surface of prepared coatings were assessed to examine the impact of the dopamine modification reaction on the behavior of water droplets on their surface. In this regard, the onset temperature of ice formation for a 5 mg water droplet on the coatings’ surface was measured by Differential Scanning Calorimetry (DSC) [7]. As illustrated in Figure 9, the onset temperature of the ice nucleation was reduced from −13.6 °C to −19.7 °C following the dopamine modification of the epoxy resin. These results were corroborated by the freezing delay time of water droplets on the surface of coatings at −20 °C. Figure 10 illustrates the behavior of a 4 μL water droplet on the surface of both pristine and modified coatings at various stages: initial dripping, freezing onset, and complete freezing, along with their corresponding times. Consistent with the DSC results, the freezing delay time significantly increased from 17 s for the unmodified coatings to 1316 s for the modified coatings at −20 °C. This substantial increase in freezing delay time for the dopamine-modified coatings at −20 °C aligns well with their ice nucleation temperature (−19.7 °C), allowing the supercooled water droplets to remain in their metastable state for a long time below the equilibrium freezing temperature (0 °C) on the surface. In contrast, water droplets froze much more rapidly on the pristine epoxy coating due to the evaluation temperature (−20 °C) being considerably lower than its ice nucleation temperature (−13.6 °C) [53].

Given that the coating surface is an external agent influencing ice nucleation in the water droplet, a heterogeneous ice nucleation process is expected [55]. Therefore, the characteristics of the surface and its interface with water play a crucial role in the ice nucleation behavior [56]. The delayed ice nucleation observed on the surface of the modified coating can be attributed to the strong hydrogen-bonding interactions between water molecules and bidentate hydroxyl groups of catechol units [40,57]. These hydrogen-bonded molecules can form a thin layer (nm scale) of water on the surface with reduced entropy and enhanced viscosity capable of maintaining a non-ordered liquid-like molecular structure even at sub-zero temperatures. This layer is commonly referred to as the “quasi-liquid layer” (QLL) [58]. Considering the heterogeneous nucleation theory for the growth of an ice embryo on the surface of coating within the super-cooled water droplet, the presence of this interfacial QLL between the ice embryo and the surface is proven to influence the ice–water contact angle (θ_IW_), as well as the self-diffusion of water molecules across the interface, both favoring the extension of the freezing delay time of the surface [56,59].

#### 3.4.2. Non-Frozen Quasi-Liquid Layer (QLL) Characterization

**Figure 11:**
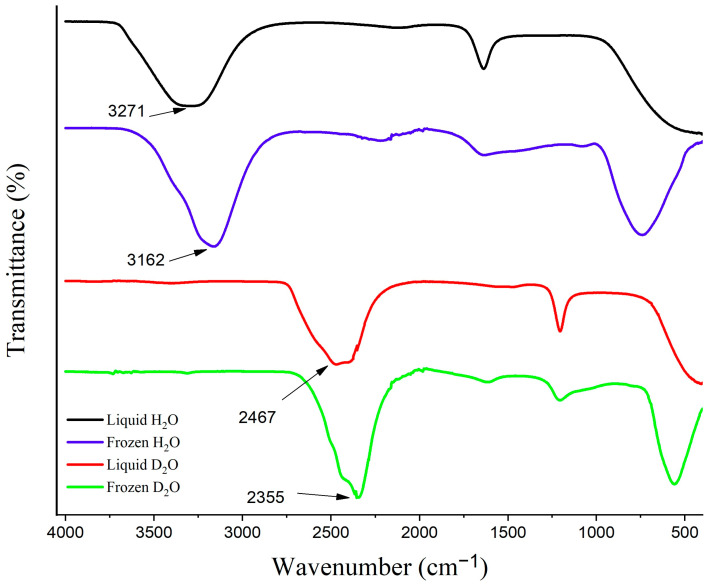
The FTIR spectra of H_2_O and D_2_O before and after freezing.

ATR-FTIR spectroscopy served as a powerful and convenient method to confirm the presence and analyze the behavior of the QLL in the present work. The evanescent wave of the reflected IR beam was utilized as a sensitive probe for studying the functional groups situated near the ATR crystal-sample interface [60]. This method was selected based on the temperature-dependent behavior of the OH stretching absorbance band of H_2_O (3307 cm^−1^), which shifts to a lower frequency (3162 cm^−1^) and becomes sharper upon freezing (Figure 11). The FTIR spectra of D_2_O before and after freezing are also presented in Figure 11 presenting the favorable freezing shift of the O-D stretching absorbance band from 2467 cm^−1^ to 2355 cm^−1^.

**Figure 12:**
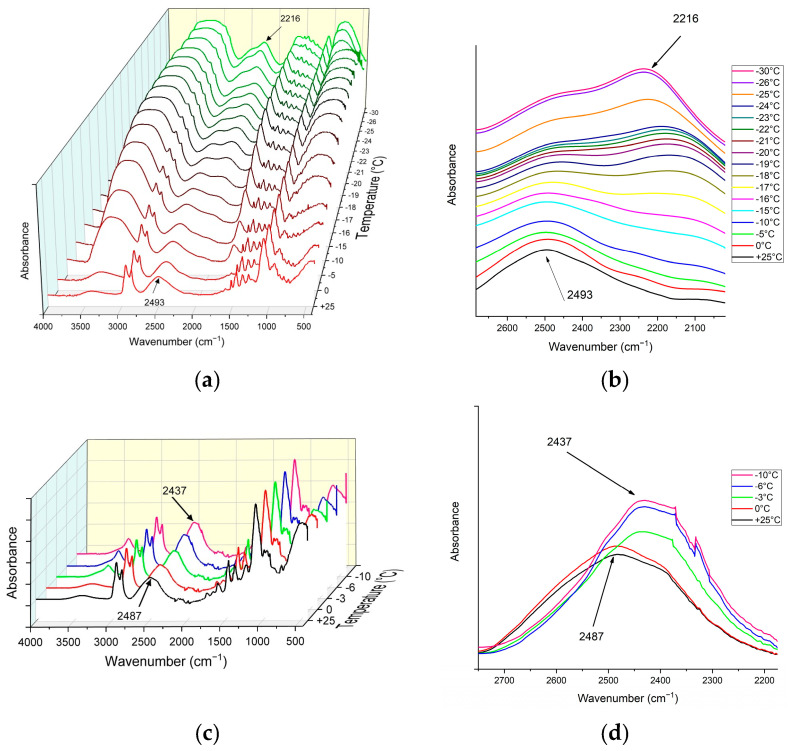
ATR-FTIR spectra of the epoxy/D_2_O coatings at various temperatures during the cooling process. (**a**,**b**): Dopamine-modified epoxy coating/D_2_O, (**c**,**d**): pristine epoxy coating/D_2_O.

Figure 12 illustrates the ATR-FTIR spectra of the Epoxy/D_2_O coatings at various temperatures during the cooling process. As can be seen in Figure 12a,b for the dopamine-modified epoxy coating, the O-D stretching band of liquid D_2_O at 25 °C appears at 2493 cm^−1^, consistent with bulk D_2_O, albeit with a slight shift likely due to interactions between the coating and D_2_O. Upon complete freezing at −26 °C, this peak shifts to 2216 cm^−1^, indicative of the O-D stretching band in solid-state D_2_O, accompanied by a gradual decrease in intensity at 2493 cm^−1^ and an increase at 2216 cm^−1^. The presence of the liquid O-D stretching band within the temperature range of D_2_O’s melting point (3.8 °C) and −26 °C suggests the existence of surficial absorbed D_2_O as a QLL in the supercooled state. Furthermore, the peak positions and intensities remain relatively unchanged from −26 °C to −30 °C, suggesting the freezing of QLL at temperatures below −26 °C. This temperature dependency is evident in Figure 12a,b. It is noteworthy that during the heating process, the O-D stretching band remained at the shifted position (2216 cm^−1^), with a shift back to the original liquid state position (2493 cm^−1^) observed within the 0 °C to 4 °C temperature range, relative to the melting point of D_2_O (3.8 °C) [60]. The shift in the O-D stretching band resulting from the D_2_O phase change was observed for the pristine epoxy coating within the 0 °C to −10 °C temperature range, with the spectrum remaining unchanged at temperatures lower than −6 °C, indicating the freezing of surficial absorbed D_2_O (Figure 12c,d). This discrepancy in the temperature of peak shift in the O-D stretching band, indicative of the phase change of D_2_O, between the pristine and dopamine-modified epoxy coatings can be attributed to the presence, as well as the higher content/thickness, of the QLL on the surface of the dopamine-modified coating and its interactions with the coating. The QLL interactions can suppress the formation and growth of ice crystals within the interface by preventing water molecules from organizing into the ordered structure of ice [58].

The Wilson–Frenkel law, utilized for measuring crystallization velocity, has been applied to investigate the kinetics of ice crystal growth within the quasi-liquid layer (QLL) [61]. This law is described by Equation (2):

(2) $$\nu_{s}=\beta_{T}(T_{m}-T),$$

where *v_s_*, *β_T_*, *T_m_*, and *T* represent the step-advancing velocity of the melt growth, the kinetic coefficient, the melting temperature of ice, and the actual temperature, respectively. The kinetic coefficient can be expressed as described by Equation (3):

(3) $$\beta_{T}=\frac{a^{2}L}{2\pi Rl_{0}\tau T_{m}T}exp(-\frac{L}{{RT}_{m}}),$$

where a represents the lattice constant, $l_{0}$ is the mean kink spacing in the step, *L* is the latent heat of fusion, *R* is the gas constant, and τ denotes the characteristic time required for a molecule in the liquid phase to incorporate into a crystal. The inverse relationship between *β_T_* and *τ* provides insight into the impact of molecular interactions on the growth rate of ice crystals within the QLL. This inverse correlation is due to the fact that the process of integrating molecules into the crystal lattice relies on material transport, which is governed by the translational diffusion of individual molecules [62]. For water, translational diffusion is directly related to the duration needed for hydrogen bond breakage [63]. Furthermore, the disorganized structure of water molecules within the QLL, which does not align with crystal symmetry, must be disrupted for a liquid molecule to integrate into the crystal lattice. Therefore, τ can be interpreted as the time required for hydrogen bonds of water molecules in the QLL to break [61]. Consequently, the observed freezing delay for dopamine-modified coatings can be attributed to their longer hydrogen bonding lifetime (as predicted by Bell’s bond lifetime theory [30]), which delays ice nucleation at super-cooled temperatures.

#### 3.4.3. Ice Adhesion Strength and Durability

**Figure 13:**
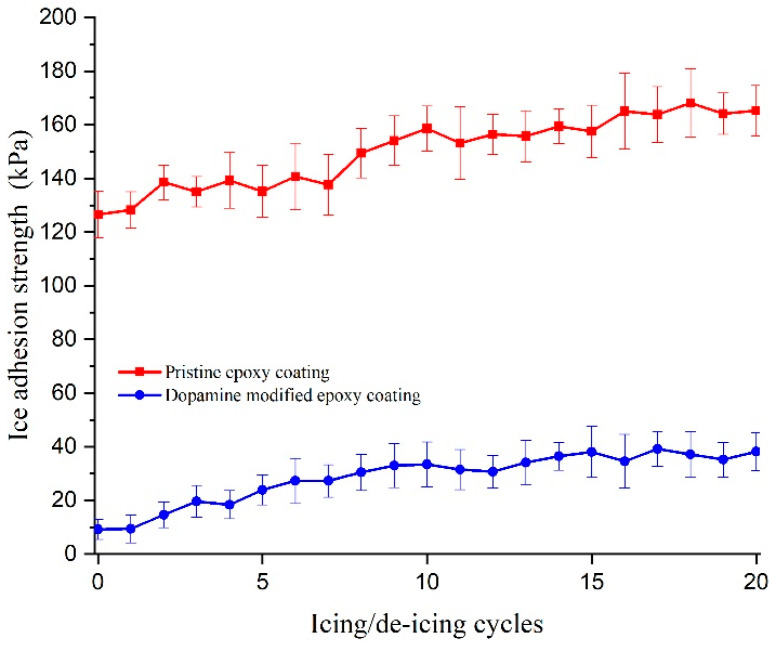
Push-off ice adhesion strength of the pristine and modified epoxy coatings for 20 consecutive icing/de-icing cycles.

The icephobic properties of the prepared coatings were evaluated using two different techniques. The first technique involved measuring the push-off adhesion strength, which was calculated by dividing the force required to detach a bloc of non-impact bulk ice from the coating by the area of the ice cylinders in contact with the surface. This test was performed on five samples of each coating, and the average adhesion strength was reported. Furthermore, to assess the durability of the anti-icing approach, the experiment was repeated consecutively for 20 icing/de-icing cycles on each sample, and the average ice adhesion strength was recorded. The results for the push-off ice adhesion strength are illustrated in Figure 13. The results demonstrate that the pristine epoxy coating exhibited an ice adhesion strength of 126.5 ± 8.5 kPa during the first icing/de-icing cycle, whereas the dopamine-modified epoxy coating displayed a significantly reduced ice adhesion strength of 9.2 ± 3.6 kPa under the same conditions. Concerning the fact that no other alterations were made to the formulation or surface of the coatings, this substantial reduction in ice adhesion strength can be attributed to the modification of the molecular structure through the introduction of dopamine molecules. The strong hydrogen bonding between surficial catechol units and water molecules can promote a quasi-liquid layer (QLL) on the surface of the modified coating at sub-zero temperatures [57]. This QLL acts as a lubricating layer that facilitates the ice detachment from the surface [25,64]. These findings align with the observed trends in the water contact angle (WCA) results, indicating an enhanced hydrophilic behavior for the modified coating [7]. Additionally, the ice adhesion strengths calculated for both the pristine and modified coatings during the remaining icing/de-icing cycles show a slightly increasing trend. This slight increase was deemed negligible, as it could be attributed to variations in surface roughness and was not substantial enough to be considered a significant alteration in the material’s behavior.

**Figure 14:**
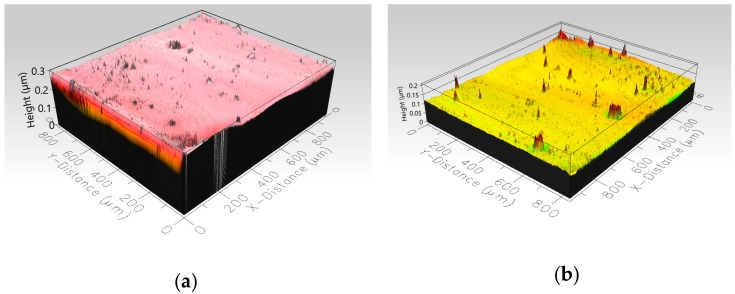

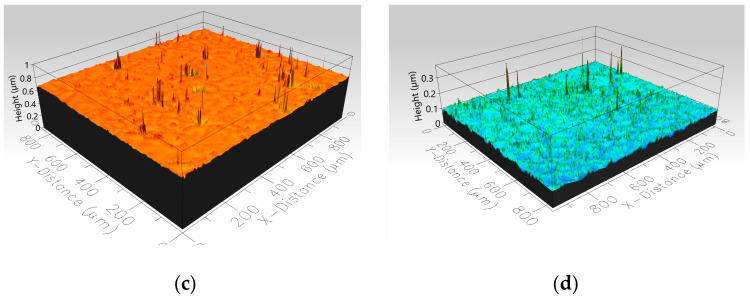
Surface topography before and after 20 icing/de-icing cycles for the pristine (**a**,**b**) and modified (**c**,**d**) epoxy coatings.

The surface topography of the samples was examined before and after conducting the 20 icing/de-icing cycles, as depicted in Figure 14. The analysis of the surface structure confirmed that there were no significant changes in the coatings’ surface topography following repeated ice detachment. The root mean square height (Sq), which serves as an indicator of the area roughness, was measured before and after the tests as 0.011 µm and 0.018 µm for the pristine epoxy coating and as 0.020 µm and 0.015 µm for the modified epoxy coating, respectively. These results suggest that the ice detachment process did not significantly alter the surface structure of the coatings. The Sq values before and after testing remained comparable, indicating the preservation of the coatings’ initial surface topography.

To further validate the push-off results and simulate the coatings’ performance under atmospheric icing conditions, a centrifugal adhesion test (CAT) was conducted. In this test, instead of using non-impact bulk ice, supercooled water microdroplets were precipitated on the coated beams at −10 °C to simulate glaze ice precipitation on their surfaces. The beams were then accelerated in a centrifuge to determine the rotational speed at which the precipitated ice would shed off due to centrifugal force.

Table 3 provides the properties of the CAT samples and the calculated ice adhesion strengths for both the unmodified and modified epoxy coatings. Six samples were tested for each type of coating. Based on the results, ice detachment occurred significantly faster (11 s vs. 27 s) and subsequently at a much lower rotational speed (3215 rpm vs. 8029 rpm), resulting in a lower ice detachment centrifugal force (99 N vs. 596 N) for the dopamine-modified coatings compared to the pristine epoxy coatings. As a result, the average ice adhesion strengths were determined to be 85 kPa for the dopamine-modified epoxy coatings and 617 kPa for the pristine epoxy coatings. These results align with the push-off ice adhesion measurements and strongly support the effectiveness of the applied approach in developing icephobic epoxy-based coatings, which are typically known for their weak anti-icing performance [28,65].

Furthermore, the Adhesion Reduction Factor (*ARF*) was measured to be 7.2 using Equation (4):

(4) ARF=τcepτdep,

where $\tau_{cep}$ represents the ice adhesion strength of the control (unmodified) epoxy coating, and $\tau_{dep}$ represents the ice adhesion strength of the dopamine-modified epoxy coating. The obtained *ARF* value provides a quantifiable measure of the improvement in ice adhesion resistance achieved through dopamine modification.

**Figure 15:**
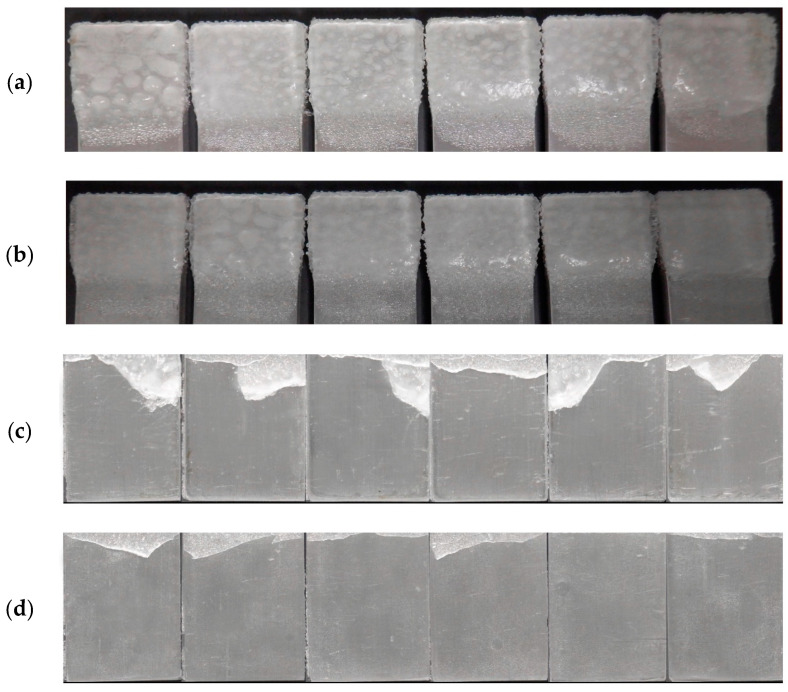
Images of ice-precipitated coatings before and after the CAT test for the beams coated with pristine (**a**,**c**) and modified (**b**,**d**) epoxy coatings.

Furthermore, the visual evidence supporting the calculations and observations is provided by the photographs of the ice-precipitated beams before and after the CAT test, as depicted in Figure 15. These images serve to validate the results obtained from the calculations, clearly demonstrating a higher amount of ice detachment from the surface of the modified epoxy coatings compared to the unmodified coatings. The visual comparison highlights the improved ice-releasing properties of the modified coatings, further reinforcing the efficacy of the dopamine modification in enhancing the anti-icing performance.

## 4. Conclusions

In conclusion, this feasibility study explored the potential of dopamine-modified epoxy coatings for anti-icing applications. The incorporation of dopamine into the epoxy resin resulted in enhanced hydrophilicity and the formation of aqueous lubricating layers on the surface. The modification reaction was successfully confirmed through FTIR and NMR analysis. A comprehensive characterization of the coatings was conducted, including X-ray Photoelectron Spectroscopy (XPS), wettability measurements, surface roughness analysis, hardness, adhesion strength, tensile strength, and abrasion resistance. The findings indicated that the introduction of dopamine led to an increase in the aromatic hydroxyl groups on the coating surface, providing higher hydrophilicity as a means of inducing water QLL on it. It should be noted that the modification reaction led to a decrease in hardness and stiffness, while increasing flexibility, due to a reduction in crosslink density. Despite these changes in mechanical properties, the dopamine-modified coatings exhibited strong performance compared to other icephobic coatings reported in the literature. The presence of QLL on the surface of the modified coating was confirmed by low-temperature ATR-FTIR spectroscopy, which was addressed as the main reason for its notable ice nucleation delay time and temperature. The ice adhesion strength was evaluated using push-off and centrifuge adhesion tests, and the durability of the icephobic properties was assessed through repeated icing/de-icing cycles. The results showed a very good ice adhesion reduction factor of 7.2, confirming the feasibility of dopamine-modified epoxy coatings as effective icephobic materials and demonstrating acceptable mechanical durability and reduced ice adhesion strength.

## Figures and Tables

**Table 1:** Relative area contribution of different functional groups measured by Casa XPS.

		Pristine Epoxy Coating	Dopamine-Modified Epoxy Coating
	C–C	83.65%	56.41%
C1s	C–O	16.35%	26.34%
	C=C	--	17.25%

**Table 2:** Mechanical properties of the prepared epoxy coatings *.

	Pristine Epoxy Coating	Dopamine-Modified Epoxy Coating
Tensile strength (MPa)	20.8 ± 1.6	9.6 ± 0.9
Elongation at break (%)	9 ± 3	45 ± 8
Elastic modulus (MPa)	576 ± 38	215 ± 17
Hardness (s)	255 ± 19	130 ± 7.5
Abrasion resistance: wear index (mg/cycle)	32 ± 3	43 ± 4
Adhesion strength (MPa)	3.34 ± 0.38	5.74 ± 0.43

* Data are summarized as “mean ± standard deviation” for 5-time test replications.

**Table 3:** CAT results for the pristine and modified epoxy coatings.

	Product and Beam #	Clean Surface (±70 mm^2^) *	Ice Mass after Icing (±0.02 g)	Ice Mass Detached (±0.02 g)	% of Ice Mass Detached	Time (s)	Speed (±25 RPM)	Force (N)	Bulk Shear Stress (±50 kPa)
Control (pristine) epoxy coatings: CEP	CEP.1	888	5.41	4.93	91	28	8374	592	666
	CEP.2	945	5.78	5.48	95	27	8092	611	646
	CEP.3	963	5.72	5.31	93	27	8070	588	610
	CEP.4	1041	5.92	5.77	97	28	8368	681	654
	CEP.5	934	5.91	5.48	93	25	7516	528	565
	CEP.6	1029	5.88	5.66	96	26	7754	574	558
		Average				27	8029	596	617
		Standard deviation				1	340	50	47
		% Variation				4%	4%	8%	8%
Dopamine-modified epoxy coatings: DEP	DEP.1	1053	5.63	5.52	98	10	3147	92	87
	DEP.2	1213	5.69	5.61	99	11	3224	97	80
	DEP.3	1199	5.86	5.78	99	10	3144	95	79
	DEP.4	1122	5.83	5.76	99	11	3280	104	92
	DEP.5	1301	5.87	5.83	99	11	3190	97	75
	DEP.6	1122	6.05	5.97	99	11	3307	109	97
		Average				11	3215	99	85
		Standard deviation				0	68	6	9
		% Variation				2%	2%	6%	10%

* Clean surface area values are measured by image analysis of the sample photos taken after the CAT test.

## Data Availability

The data are contained within the article.
